# Vaginal delivery provides skin colonization resistance from environmental microbes in the NICU

**DOI:** 10.1002/ctm2.1506

**Published:** 2023-12-06

**Authors:** Prem Prashant Chaudhary, Brynn O'Laughlin, Purnima S. Kumar, Shareef M. Dabdoub, Shira Levy, Ian A. Myles, Suchitra K. Hourigan

**Affiliations:** ^1^ Epithelial Therapeutics Unit National Institute of Allergy and Infectious Diseases National Institutes of Health Bethesda Maryland USA; ^2^ Clinical Microbiome Unit National Institute of Allergy and Infectious Diseases National Institutes of Health Bethesda Maryland USA; ^3^ Department of Periodontics and Oral Medicine University of Michigan School of Dentistry Ann Arbor Michigan USA; ^4^ Division of Biostatistics and Computational Biology College of Dentistry University of Iowa Iowa City Iowa USA

Dear Editor,

Here, we present novel data suggesting there is an increased sharing of the environmental microbiome on the skin in infants delivered by cesarean section (CS) compared to vaginal delivery (VD) in the neonatal intensive care unit (NICU).

It has previously been shown that delivery mode is a major factor influencing early life skin bacterial colonization.^1^, ^2^, ^3^ Furthermore, skin microbiome differences by delivery mode correlate with variations in later skin barrier function and possibly health outcomes.^2^ Although reports vary, CS delivery has been associated with an increased risk of developing atopic dermatitis compared to VD in a recent large meta‐analysis.^4^ Additionally, variations in skin microbiome between infants delivered at different hospitals have been theorized to be due to variances in the microbes of the hospital environment.^2^ Building on this, we hypothesized that the overall low skin microbial load in CS infants^5^ would provide less resistance to acquisition and colonization by environmental bacteria when compared to VD infants.

To address this, shotgun metagenomic sequencing was performed on skin swabs collected from behind the ear of 73 infants (16 delivered vaginally, 57 by CS) housed in the NICU during the first week of life as previously described.^6^ Environmental swabs from different areas of the NICU were also collected. Infants who were in the NICU were selected as they were postulated to potentially acquire more environmental bacteria due to a prolonged stay in the hospital and limited skin‐to‐skin contact with family members,^7^ making them the ideal cohort to preliminarily investigate this hypothesis. Differences in the skin microbiome between delivery mode and the environment were compared. SourceTracker v0.9.5 was applied to estimate the proportion of environmental bacterial taxa in the skin microbiome.^8^ See Supplemental Data for detailed methods.

CS infants were more likely to receive peripartum antibiotics (*p* = .002) and be female (.021) than VD infants (Table S1). There were no differences in gestational age, infant antibiotic use, race or ethnicity between delivery modes.

Lower alpha diversity was observed in VD compared to CS (Shannon diversity index, *p* = .002, Figure 1A) with a clear difference in microbial composition between delivery modes (PERMANOVA, *p* = .001, Figure 1B). There were 86 significant differentially abundant bacterial genera between delivery modes. Notably, *Escherichia* and *Lactobacillus* were higher in VD, contrasted with increased *Pseudomonas*, *Staphylococcus* and *Acidovorax* in CS (Figure 1C). *Staphylococcus* and *Acidovorax* were the most distinguishing genera between delivery modes (Figure 1D). Level 4 KEGG analysis revealed lower Shannon diversity in VD (Figure 1E) with several differentially abundant pathways identified between delivery modes (Figure 1F), many involving lipid and antibiotic metabolism. Of potential interest, there was upregulation of fatty acid synthase in vaginal deliveries, with short chain fatty acids thought to have a role in the immune tolerance of the skin.^9^

**FIGURE 1:**
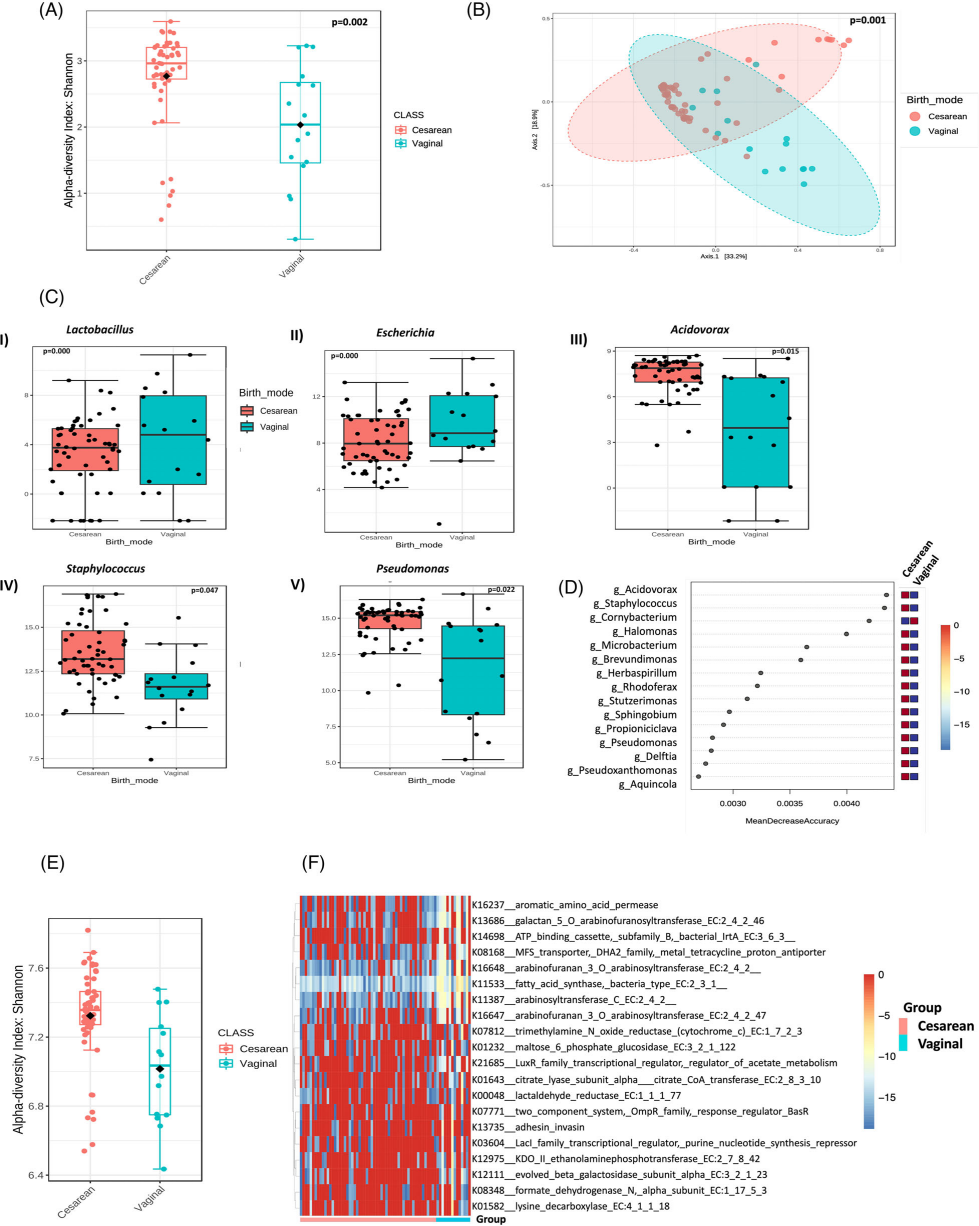
Skin microbiome differences between cesarean section (CS) and vaginal delivery (VD) infants. (A) Alpha diversity (Shannon index) of the skin microbiome between CS and VD infants. (B) principal coordinate analysis plot of the Bray‐Curtis dissimilarity distances between the microbiomes of the CS and VD infants. (C) Bar plots showing differences between CS and VD infants for the genera: *Lactobacillus*, *Escherichia*, *Acidovorax*, *Staphylococcus* and *Pseudomonas*. (D) Random forest plot showing the most distinguishing taxa at the genus level between delivery modes. (E) Differences in alpha diversity of functional pathways (level 4 KEGG) between CS and VD. (F) Heatmap plot showing the top 20 functional enzymes based on their log2 fold change values (level 4 KEGG).

The microbiome of the NICU environment clustered with the skin bacteria in those delivered by CS (*p* = .001, Figure 2A). SourceTracker analysis demonstrated a significantly greater sharing of environmental microbes in CS compared with VD infants (*p* = .006). These organisms predominantly were sourced from a nurses’ station wash sink (*p* = .007, Figure 2B). The predominant taxa shared between the skin and wash sink belonged to the genus *Pseudomonas* (Figure 2C). There was also sharing of Staphylococcus *aureus* and species belonging to the genera *Corynebacterium* and *Cutibacterium*, each of which has previously been linked to psoriasis acne, and atopic dermatitis.^10^

**FIGURE 2:**
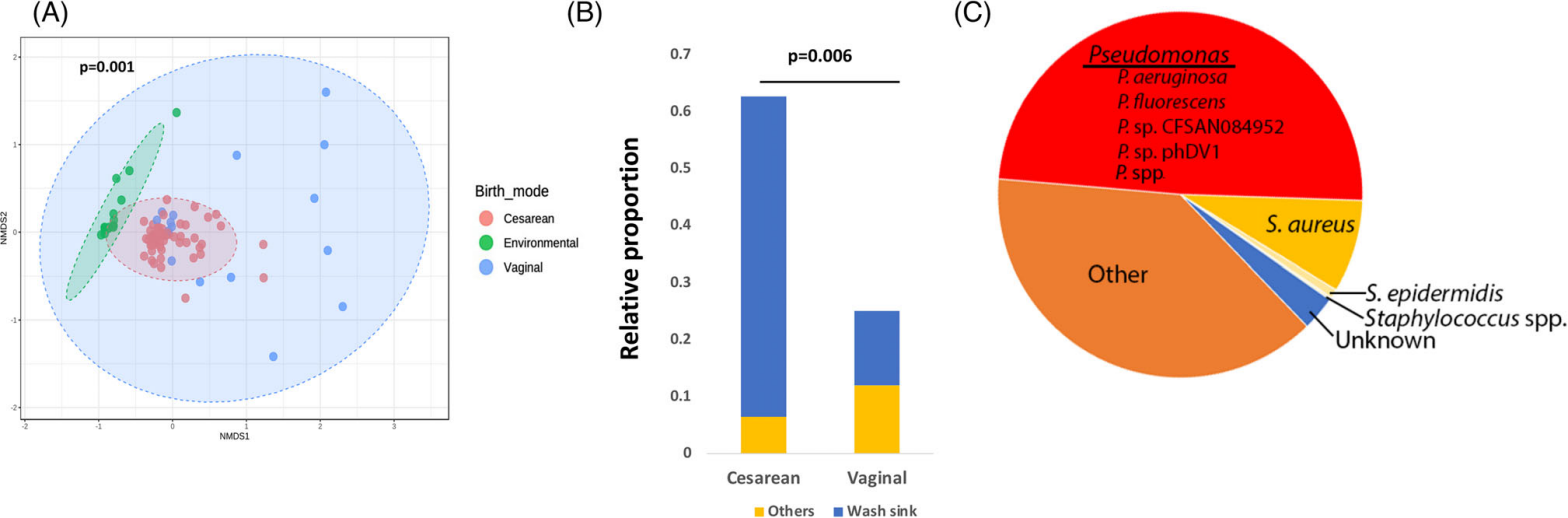
Sharing of the environmental microbiome by delivery mode. (A) Non‐metric multidimensional scaling plot visualizing the Bray‐Curtis dissimilarity matrix of the skin microbiome by delivery mode compared with environmental samples. (B) The relative proportion of environmental microbiome sources with the skin microbiome of subjects in those born by cesarean section (CS) versus vaginal delivery (VD). (C) Pie chart showing the most common taxa shared between the environment and infants’ skin.

In conclusion, there was increased sharing of the environmental microbiome on the skin of babies born by CS versus VD. We speculate that CS infants exhibited higher sharing of the environmental microbiome due to being closer to ‘sterile’ when delivered (as opposed to VD infants who are initially colonized predominantly by maternal vaginal and fecal microbes,^1^ as evidenced by higher levels of *Escherichia* and *Lactobacillus* in our study), thus having less colonization resistance and allowing opportunistic colonization by the environment. It is also possible that the higher levels of perinatal antibiotics in the CS group might contribute to the establishment of the pioneer species, by potentially reducing skin bacterial load further and allowing for more opportunistic colonization. It is difficult to separate this from the effects of CS, as perinatal antibiotics are received in all CS deliveries. Future studies looking at colonization between vaginal deliveries with and without perinatal antibiotics may help gain further insight into this. It is acknowledged that these results may not be generalizable to healthy full‐term infants who are not admitted to the NICU, who have closer skin‐to‐skin contact with family members and different environmental exposures; this warrants further assessment. In addition, the overall sample size is limited especially for vaginal deliveries, and further investigation should include a larger cohort. Moreover, examining the microbiome of other potential sources of colonization such as the caretakers’ microbiome and breast milk microbiome may help to tease apart other contributors. Lastly, the differences in functional pathways found between delivery modes are of potential interest with exploration in larger studies needed to see if this may have a lasting impact.

These preliminary data underscore the importance of the environment in the development of the skin microbiome. Most importantly, the impact of our findings on later health outcomes, including skin diseases associated with delivery mode, warrants further investigation.

## AUTHOR CONTRIBUTIONS

*Concept and design*: Suchitra K. Hourigan and Ian A. Myles. *Acquisition, analysis or interpretation of data*: All authors. *Drafting of the manuscript*: Suchitra K. Hourigan. *Critical revision of the manuscript for important intellectual content*: All authors. *Statistical analysis*: Prem Prashant Chaudhary, Shareef M. Dabdoub and Purnima S. Kumar. *Obtained funding*: Suchitra K. Hourigan and Ian A. Myles. *Supervision*: Suchitra K. Hourigan and Ian A. Myles. *Reviewed and approved the manuscript*: All authors.

## FUNDING INFORMATION

National Institute of Allergy and Infectious Diseases of the National Institutes of Health

## CONFLICT OF INTEREST STATEMENT

The authors have declared that no conflict of interest exists.

## ETHICS STATEMENT

The study was Institutional Review Board approved (WCG IRB 1300205), and written informed consent was obtained.

## Supporting information

Supporting Information

Table S1: Clinical and demographic features compared between infants born by Cesarean section and vaginal delivery.

## Data Availability

The data sets generated and/or analyzed in the current study are available in the NCBI SRA repository under BioProjects ID PRJNA854811 (infant data) and PRJNA417283 (environmental data).
