# Supplementary material for: Vaginal delivery provides skin colonization resistance from environmental microbes in the NICU
Source: Clin Transl Med. 2023 Dec 6;13(12):e1506. doi: 10.1002/ctm2.1506 (PMC10701179; doi:10.1002/ctm2.1506)
Supplement: Supplementary file 1 — Supporting Information [file CTM2-13-e1506-s001.docx]

**Supplemental Methods**

Subjects and samples: Neonates who spent time in the Inova Fairfax Neonatal Intensive Care Unit (NICU) were enrolled in an observational longitudinal microbiome cohort study. The study was Institutional Review Board approved (WCG IRB 1300205) and parental informed consent was obtained. Neonates were enrolled within the first week of life and had an anticipated stay in the NICU of >5 days. While in the NICU, detailed demographic and clinical data was collected along with samples for microbiome analysis (including skin swabs and stool). Specifically for the skin, a sterile cotton swab was moistened with sterile saline and rolled behind the neonate’s ear in a standardized manner. Environmental swabs were collected in a similar manner from different areas in the NICU where the infants were hospitalized, including the wash sink at the nurses’ station, the desk at the nurses’ station, the light switch and the foam hand sanitizer pump.

Skin and environmental swabs underwent DNA extraction, library prep and sequencing with CosmosID, Germantown, MD. DNA from swabs was isolated using the ZymoBIOMICS MicroPrep kit, according to the manufacturer’s protocol. DNA libraries were prepared using the Nextera XT DNA Library Preparation Kit (Illumina) and IDT Unique Dual Indexes with total DNA input of 1ng. Genomic DNA was fragmented using a proportional amount of Illumina Nextera XT fragmentation enzyme. DNA libraries were quantified using Qubit 4 fluorometer and Qubit™ dsDNA HS Assay Kit. Libraries were then sequenced on an Illumina NovaSeq 6000 platform.

After removal of human genome sequences, the taxonomic profile for bacterial counts was determined using Kraken (Version 1.2.0). Prodigal (Version 2.6.3) was used for out functional analysis gene calling, and called genes were aligned against NCBI-nr database using DIAMOND (Version 2.0.11.149). Finally, the functional analysis was performed by using MEGAN (Version 6.13.3). Both the taxonomic and functional count tables were then loaded to MicrobiomeAnalyst and after rarefying the data to the minimum library size and data scaling different statistical analysis were performed and plots were generated. Source tracking Bayesian analysis (SourceTracker v0.9.5) was used to trace the sources of the skin microbiomes to the environmental sources.
