# Supplementary material for: Vaginal delivery provides skin colonization resistance from environmental microbes in the NICU
Source: Clin Transl Med. 2023 Dec 6;13(12):e1506. doi: 10.1002/ctm2.1506 (PMC10701179; doi:10.1002/ctm2.1506)
Supplement: Supplementary file 2 — Table S1: Clinical and demographic features compared between infants born by Cesarean section and vaginal delivery. [file CTM2-13-e1506-s002.docx]

Supplemental Table 1: Clinical and demographic features compared between infants born by Cesarean section and vaginal delivery

| **Clinical factor** | **Vaginal delivery (n=16)** | **Cesarean Section (n=57)** | **P value** |
| --- | --- | --- | --- |
| **Sex :**  Male (n, %)  Female (n, %) | 14, 87.5%  2, 12.5% | 31, 55.3%  25, 44.6% | **0.021** |
| **Gestational Age (mean, range)** | 32.4, 23-40 | 32.2, 24-39 | 0.839 |
| **Ethnicity:**  Non Hispanic or Latino (n, %)  Hispanic or Latino  Unknown | 10, 62.5%  3, 18.8%  3, 18.8% | 41, 73.2%  5, 8.8%  10, 17.5% | 0.484 |
| **Race:**  White or Caucasian  Black or African American  Asian  Other  Unknown | 6, 37.5%  3, 18.8%  5, 31.3%  0, 0%  2, 12.5% | 38, 67.9%  4, 7.1%  7, 12.5%  4, 7.1%  3, 5.4% | 0.057 |
| **Maternal perinatal antibiotics**  Yes (n, %)  No (n, %) | 12, 75%  4, 25% | 56, 100%  0, 0% | **0.002** |
| **Infant antibiotics prior to skin swab** (n, %)  Yes  No | 0, 0%  16, 100% | 0, 0%  56, 100% | 1 |
| **Day of life of swab**  Mean  Range | 2.5  0-5 | 2.79  0-7 | 0.487 |
